# miRTarBase 2016: updates to the experimentally validated miRNA-target interactions database

**DOI:** 10.1093/nar/gkv1258

**Published:** 2015-11-20

**Authors:** Chih-Hung Chou, Nai-Wen Chang, Sirjana Shrestha, Sheng-Da Hsu, Yu-Ling Lin, Wei-Hsiang Lee, Chi-Dung Yang, Hsiao-Chin Hong, Ting-Yen Wei, Siang-Jyun Tu, Tzi-Ren Tsai, Shu-Yi Ho, Ting-Yan Jian, Hsin-Yi Wu, Pin-Rong Chen, Nai-Chieh Lin, Hsin-Tzu Huang, Tzu-Ling Yang, Chung-Yuan Pai, Chun-San Tai, Wen-Liang Chen, Chia-Yen Huang, Chun-Chi Liu, Shun-Long Weng, Kuang-Wen Liao, Wen-Lian Hsu, Hsien-Da Huang

**Affiliations:** 1 Institute of Bioinformatics and Systems Biology, National Chiao Tung University, Hsinchu, 300, Taiwan; 2 Graduate Institute of Biomedical Electronics and Bioinformatics, National Taiwan University, Taipei, 106, Taiwan; 3 Department of Biological Science and Technology, National Chiao Tung University, Hsinchu, 300, Taiwan; 4 Center for Bioinformatics Research, National Chiao Tung University, Hsinchu, 300, Taiwan; 5 Clinical Research Center, Chung Shan Medical University Hospital, Taichung, 402, Taiwan; 6 Institute of Population Health Sciences, National Health Research Institutes, Miaoli, 350, Taiwan; 7 Interdisciplinary Program of Life Science, National Tsing Hua University, Hsinchu, 300, Taiwan; 8 Institute of Molecular Medicine and Bioengineering, National Chiao Tung University, Hsinchu, 300, Taiwan; 9 Degree Program of Applied Science and Technology, National Chiao Tung University, Hsinchu, 300, Taiwan; 10 Gynecologic Cancer Center, Department of Obstetrics and Gynecology, Cathay General Hospital, Taipei, 106, Taiwan; 11 Institute of Genomics and Bioinformatics, National Chung Hsing University, Taichung, 402, Taiwan; 12 Department of Obstetrics and Gynecology, Hsinchu Mackay Memorial Hospital, Hsinchu, 300, Taiwan; 13 Mackay Medicine, Nursing and Management College, Taipei, 112, Taiwan; 14 Department of Medicine, Mackay Medical College, New Taipei City, 252, Taiwan; 15 Institute of Information Science, Academia Sinica, Taipei, 115, Taiwan; 16 Department of Biomedical Science and Environmental Biology, Kaohsiung Medical University, Kaohsiung, 807, Taiwan

## Abstract

MicroRNAs (miRNAs) are small non-coding RNAs of approximately 22 nucleotides, which negatively regulate the gene expression at the post-transcriptional level. This study describes an update of the miRTarBase (http://miRTarBase.mbc.nctu.edu.tw/) that provides information about experimentally validated miRNA-target interactions (MTIs). The latest update of the miRTarBase expanded it to identify systematically Argonaute-miRNA-RNA interactions from 138 crosslinking and immunoprecipitation sequencing (CLIP-seq) data sets that were generated by 21 independent studies. The database contains 4966 articles, 7439 strongly validated MTIs (using reporter assays or western blots) and 348 007 MTIs from CLIP-seq. The number of MTIs in the miRTarBase has increased around 7-fold since the 2014 miRTarBase update. The miRNA and gene expression profiles from The Cancer Genome Atlas (TCGA) are integrated to provide an effective overview of this exponential growth in the miRNA experimental data. These improvements make the miRTarBase one of the more comprehensively annotated, experimentally validated miRNA-target interactions databases and motivate additional miRNA research efforts.

## INTRODUCTION

MicroRNAs (miRNAs) are a class of endogenous non-coding RNAs with ∼22 nucleotides (nt) that play important roles at the post-transcriptional level in animals and plants (1). The mechanistic model of miRNAs regulates gene expression either by repressing mRNA translation or by inducing mRNA degradation by partial complementarity binding with target sequences (2). Importantly, several miRNAs were found to have a critical role in regulating many physiological processes, such as the cell-cycle (3), cell growth, development, differentiation (4) and apoptosis (5), and pathological processes, such as those associated with various cancers (6). Additionally, miRNAs may be good candidate for the early detection or prognosis biomarkers for various diseases (7).

miRNA deregulation leads to a number of clinically important diseases, ranging from myocardial infarction to various types of cancer (6). Many freely available and web-based miRNA-related database systems have been developed for analyzing miRNAs and their target genes. miRBase (8) is the largest web-accessible repository that provides integrated interfaces for comprehensive microRNA nomenclature, sequence and annotation data. miRNA databases, such as microRNA.org (9), miRGator (10), miRDB (11) and miRNAMap (12) integrate target prediction programs to identify miRNA target-interactions (MTIs). Several other miRNA databases have been developed to provide evidence for experimentally validated miRNAs and their target genes. DIANA-TarBase (13), similar to our database hosts detailed information concerning each miRNA-gene interaction, ranging from miRNA- and gene-related facts to information that are specific to their interactions, experimental validation methodologies and their outcomes. HMDD (14) is a database that collects experimentally supported human microRNA and disease associations and integrates miRNA-disease association data from genetics, epigenetics, circulating miRNAs and MTIs. miRecords (15) contains manually curated, experimentally validated and predicted miRNA targets from 11 established miRNA target prediction programs. miR2Disease (16) is a manually curated database, providing a comprehensive resource for microRNA deregulation in various human diseases with brief descriptions of microRNA-disease relationships, microRNA expression patterns, microRNA expression detection methods, and experimentally verified target genes for microRNA, as well as literature references. miRWalk (17) is a comprehensive database that provides predicted as well as validated miRNA binding site information concerning on miRNAs in humans, mice and rats through an automated and supports extensive text-mining to extract validated information on miRNAs. The DIANA-LncBase (18) is a database of miRNA-lncRNA-putative functional interactions and provides comprehensive annotations of miRNA targets on lncRNAs. miRGate (19) contains novel computationally predicted miRNA–mRNA pairs as well as experimentally validated data from four well known databases. Despite the large number of available databases of MTIs, microRNA target gene related research has greatly increased in recent years, so an easily accessible centralized information repository of experimentally validated microRNA-target interactions that can be updated over the long-term must be developed.

Computational prediction programs constitute the first means of identifying miRNA targets. These programs are usually based on the phylogenetically conserved complementarity of miRNAs to their potential target genes (20). However, perfect seed pairing may not be an entirely accurate predictor (21). Experimental research must still validate the interaction between the microRNA and its target sites to elucidate the functions of microRNA. Therefore, the interaction of a miRNA with its target gene is typically verified by specific experimental validation that involves well-established techniques, such as qRT-PCR, luciferase reporter assay and western blot (22). Western blot and qRT-PCR measure the expressions level of protein and the mRNA level, respectively. Reporter assays are reliable methods for elucidating the direct interaction between microRNA and its target gene that are based on the binding of a given miRNA to its specific mRNA target site to repress the production of the reporter protein, reducing activity or expression (23). Northern blot analysis, ribonuclease protection assay or *in situ* hybridization are commonly performed to elucidate the reciprocal expression of predicted miRNA and mRNA target genes (24). Also, proteomic stable isotope labeling with amino acids in culture (SILAC) or pulsed SILAC (pSILAC) (25) has been developed to measure global changes in the proteome following the over-expression or silencing of miRNA. Recently, numerous miRNAs and their associated targets have been identified by high-throughput sequencing, such as CLIP-seq, PAR-CLIP and CLASH.

To facilitate the study of microRNA targets and its importance in many physiological processes, we previously developed the miRTarBase of manually curated and experimentally validated MTIs from the literature. This work provides an important repository of experimentally validated MTIs with more useful annotations and visualization data for biologists.

## IMPROVEMENTS

**Figure 1.**
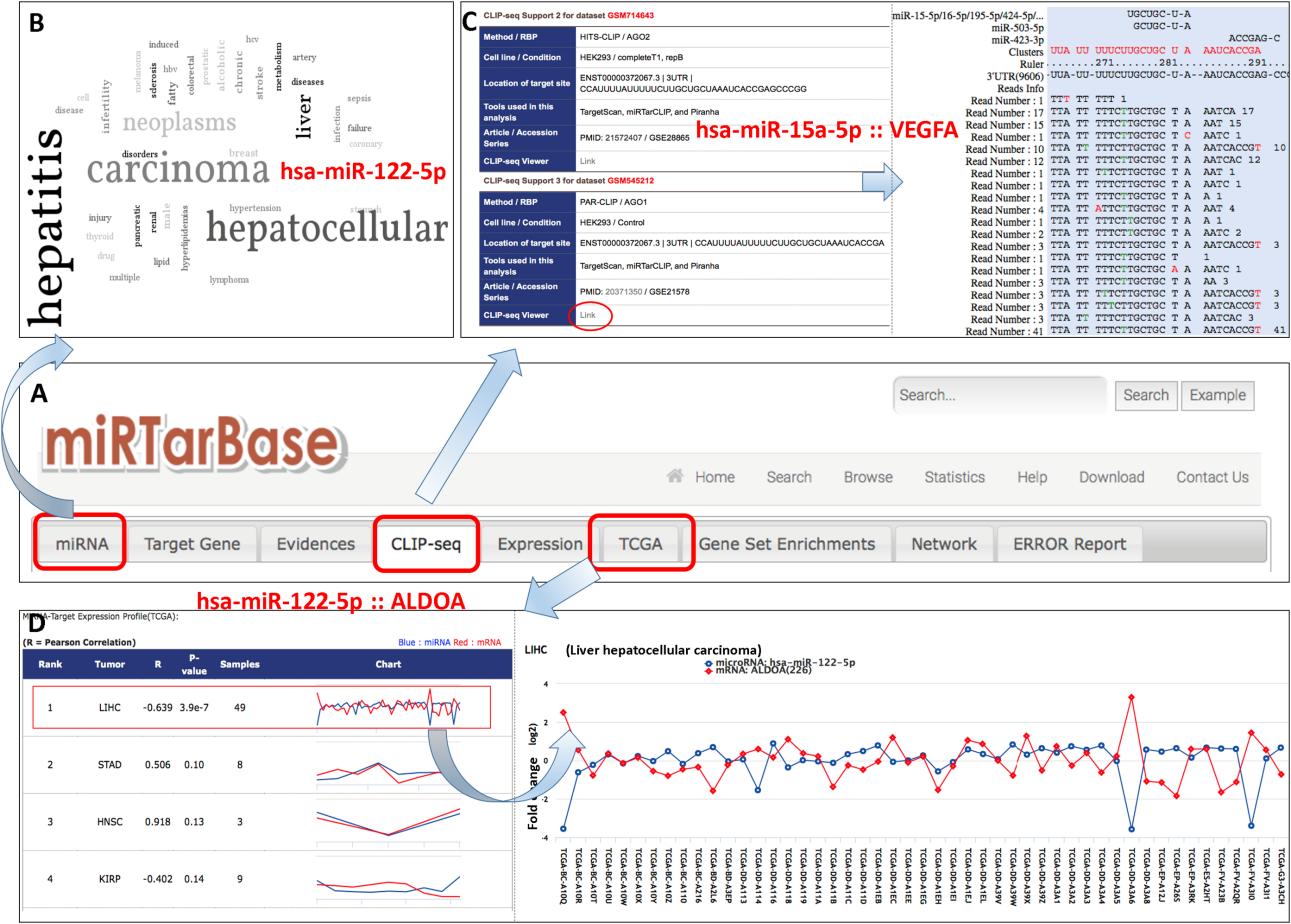
Snapshot of major improvements provided by miRTarBase interface. (**A**) New miRTarBase home page and three new major interfaces, including miRNA disease word cloud (**B**), miRNA-target site viewer based on CLIP-seq data (**C**) and miRNA and gene expression profiles obtained using TCGA data set (**D**).

Table 1 presents the improvements and advances that are provided by the miRTarBase 2016 update. Figure 1 displays the new features of the web interface. Major improvements over the last two years include (i) a significant increase in the number of MTIs, including both strong and limited experimental evidence, (ii) an expansion of microRNA and gene expression profiles using the The Cancer Genome Atlas (TCGA) data set, (iii) the development of an automatic text-mining system to extract miRNA-target interactions for further manual review and (iv) the creation of an enhanced web interface. The miRTarBase has been continuously maintained with periodic data updates. The details of each improvement are as follows:

**Table 1.** Advances and improvements provided by miRTarBase 6.0

Features	miRTarBase 4.5	miRTarBase 6.0
Release date	2013/11/01	2015/09/15
Curated articles	2636	4966
miRNAs	1232	3786
Target genes	17 520	22 563
Curated miRNA-target interactions	51 460	366 181
miRNA and target gene expression profiles	GEO	GEO, TCGA (571 data sets, 19 cancer type)
Text-mining technique to prescreen literature	Keyword search	NLP
Abstract annotation data for download	None	Yes
MTIs were validated by experimental technology	Reporter assay, western blot, northern blot, qRT-PCR, microarray, pSILAC, NGS (HITS-CLIP, Degradome-seq and CLASH)	Reporter assay, western blot, northern blot, qRT-PCR, microarray, pSILAC, 3′ LIFE, TRAP, NGS (HITS-CLIP, Degradome-seq, CLASH, PAR-CLIP, iPAR-CLIP).
Graphical visualization	miRNA, secondary structure, known and novel miRNA target sites, functional and nonfunctional MTIs, experimental conditions, miRNA-target network, upgrade reminder service, error report system, user feedback service, miRNA-target expression profile from GEO.	miRNA, secondary structure, known and novel miRNA target sites, functional and non-functional MTIs, experimental conditions, miRNA-target network, upgrade reminder service, user feedback service, miRNA-target expression profile from GEO and TCGA, updated error report system, enhanced search system, word cloud of miRNA-disease information, miRNA-Target site viewer using CLIP-seq data.

### Updated database content

This study reports on the expanded and more highly curated content of the miRTarBase. The current release (September 15, 2015, version 6) includes a total of 366 181 curated MTIs between 3786 miRNAs and 22 563 target genes, which were collected from 4966 articles. The number of MTIs in miRTarBase has increased ∼7-fold since the 2014 miRTarBase update (miRTarBase v4.5) (26). MTIs with different degrees of experimental support are available in miRTarBase. Table 2 shows the numbers of MTIs in miRTarBase 4.5 and miRTarBsae 6.0. Strong evidence of MTIs was curated by manually surveying the relevant literature and the limited experimental evidence that was related to CLIP-seq (HITS-CLIP, PAR-CLIP) was analyzed using the NGS data, described below.

**Table 2.** Number of miRNA-target interactions with different validation methods in miRTarBase 4.5 and miRTarBase 6.0

Features	miRTarBase 4.5	miRTarBase 6.0
MTIs Supported by strong experimental evidences		
Number of MTIs validated by ‘Reporter assay’	4109	6694
Number of MTIs validated by ‘Western blot’	2405	4580
Number of MTIs validated by ‘qPCR’	2512	4645
Number of MTIs validated by ‘Reporter assay and western blot’	1915	3854
Number of MTIs validated by ‘Reporter assay or western blot’	4563	7439
MTIs Supported by limited experimental evidences		
Number of MTIs validated by ‘Microarray’	12 547	13 587
Number of MTIs validated by ‘NGS’	31 907	348 007

#### High-throughput experimental methods for identifying miRNA-target interactions

Microarray technology provides a powerful, high-throughput platform to detect miRNA or gene expression levels and, since 2005, has often been used in genome-wide studies of miRNA or the gene expressions of case and control samples (27–29). Powerful next-generation sequencing technologies, such as small RNA-seq and RNA-seq, are also used to detect miRNA and gene expression levels (30). However, none of these methods can directly verify miRNA-target interactions. A quantitative-mass-spectrometry-based method that uses SILAC (stable isotope labeling with amino acids in cell culture) (31) and pSILAC (25) can detect genome-wide protein expressions of several thousand proteins in response to miRNA transfection or endogenous miRNA knockdown. Recently, CLIP-seq (also called HITS-CLIP) and PAR-CLIP approaches have been extensively utilized to identify MTIs. Chi *et al*. (32) were the first to use the cross-linking and immunoprecipitation approach (CLIP) with NGS techniques (HITS-CLIP) to identify MTIs. Hafner *et al*. (33) used photoactivatable-ribonucleoside-enhanced cross-linking and immunoprecipitation (PAR-CLIP) to increase the resolution of the original CLIP-seq method. To study the interactions between miRNA and mRNA more directly, two groups modified CLIP-seq methods CLASH (34) and iPAR-CLIP (35) that ligate the miRNA and mRNA sequences to produce miRNA–mRNA chimera sequences. German *et al*. (36) developed an approach called degradome-seq to detect MTIs by identifying mRNA cleavage products by the parallel analysis of RNA ends (PARE). The RNA-induced silencing complex (RISC)-mediated cleavage mechanism is uncommon in most mammals, so this method is primarily used in plants. Other high-throughput techniques including IMPACT-seq (37), Biotin miRNA tagging (22) and miTRAP (38), have been successfully used to identify MTIs.

#### Collection and analysis of published CLIP-seq data sets

In this updated version of miRTarBase, the number of miRNA-target interactions has been significantly increased through analyzing CLIP-seq approached data sets, such as HITS-CLIP and PAR-CLIP. These published CLIP-seq related data sets with Argonaute (AGO) RNA-binding proteins have been collected. The CLIP-seq NGS raw data were downloaded from the Gene Expression Omnibus (GEO), Short Read Archive (SRA), European Nucleotide Archive (ENA) and websites hosted by individual researchers. A total of 109 HITS-CLIP and 29 PAR-CLIP data were collected from 21 studies. The samples were annotated with the following categories: Data set source, Reference, RNA binding protein (RBP), Species, Accession number, NGS methods, Tissue/cell line and Treatment (Supplementary Table S1).

Six CLIP-seq studies do not provide NGS raw data but do provide MTIs in their supplementary data. Their results were directly included into our database. Most of the samples (138 samples) provide raw NGS data that were analyzed based on the miRTarCLIP pipeline (39) combined with Piranha (40) for peak calling and TargetScan (Release 7.0) (41) for target site identification. Doing so involves trimming the adapter for raw sequencing reads, removing low-quality reads, conducting cytidine-to-thymine reversion only for PAR-CLIP data, aligning reads against the reference 3′ UTR sequence, searching target site clusters and peak calling and microRNA-target interaction analysis. In comparison with previous version of miRTarBase, this version significantly increases the CLIP-seq supported miRNA-target interactions. The row ‘*Number of MTIs validated by ‘NGS’*’ in Table 2 shows around 11-fold increment of the number of miRNA-target interactions.

#### miRNA-target associated disease

Dysregulated miRNAs are reportedly associated with many diseases. Recently, studies have reported that some circulating miRNAs are biomarkers for disease diagnosis and provide clues concerning potential disease therapies. Aside from experimentally validated miRNA disease information that was curated by our group, miRTarBase integrates the data of HMDD version 2.0, miR2Disease, ExcellmiRDB (42) and miRCancer (43), to provide extensive information that concerns experimentally validated miRNA-associated diseases and the relationship between miRNA-target interactions and disease.

### Expansion of MicroRNA and gene expression profiles using TCGA data set

TCGA collects and analyzes high-quality tumor samples and provides clinical information about participants in the relevant studies, metadata about the samples, histopathology slide images and other molecular information (such as copy number variation, DNA methylation, single-nucleotide polymorphism, protein expression, DNA sequencing and mRNA/microRNA expression) that are obtained using the array-based or NGS-based technique. The correlation of the expression of miRNA with that of mRNA importantly indicates the direct targets of miRNA. Large TCGA clinical data sets are used in clinical research to develop microRNA biomarkers or to help biologists study tumor-specific miRNA regulatory pathways. starBase v2.0 (44) displays pan-cancer analysis of interactions between RNA-binding proteins and RNAs as well as miRNA-target interactions, including 6000 samples for 14 types of cancer from TCGA. Like starBase, the database herein includes expression data that match the TCGA miRNA and mRNA samples to provide clinical miRNA–mRNA expression data. The TCGA currently contains more than 9000 samples of 31 cancers. To provide more unique data types and biologically meaningful data, only sequencing data from the newest platforms (miRNA-seq from Illumina HiSeq and RNA-seq from version2 Illumina HiSeq) and only selected samples that contain tumors and normal samples are collected. Tumors for which fewer than two samples available were discarded. Therefore, 571 samples of 19 cancers were selected (Supplementary Table S2). Supplementary Figure S1 (see Supplementary Document) shows the miRNA/mRNA expression profiles for miR-122–5p and ALDOA in human cancer. Only hepatocellular carcinoma exhibits a significantly negative correlation between miR-122–5p and ALDOA (*P*-value = 3.9e−7 and R = −0.639). Interestingly, Tsai *et al*. reported that miR-122 is a tumor suppressor and targets ALDOA in hepatocellular carcinoma (45). starBase includes the fold change as a bar chart. Unlike in starBase, the correlations between miRNA and their target genes are shown herein as a line chart.

### Automatic extraction of MTIs from the literature for further manual review

Unlike databases and prediction tools, miRNA-related information is largely available as unstructured text. As shown in Supplementary Figure S2 (in the Supplementary Document), the number of PubMed query results for ‘miRNA’ in the title or abstract has grown substantially since 2000. The number of miRNA related publications is estimated to grow to over 10 000 by 2016, and manual retrieval of associations between miRNA and target gene can be labor-intensive. Therefore, a two-step MTI retrieval system is developed herein to reduce the effort for curator. First, natural language processing (NLP) techniques are used to rapidly screen a large number of PubMed-indexed studies to select the correct miRNA and target gene pairs. Additionally, all of the screened documents are manually validated to extract other key elements such as terms concerning experimental methods, cell lines and descriptions of microRNA and target gene association.

Named entity recognition (NER) and relation extraction (RE) are two important components of the proposed MTI retrieval system. NER is utilized to locate and classify elements in unstructured biomedical texts, such as the names of miRNA and target genes. Relation extraction is the other crucial component of extracting miRNA-target interactions. Accordingly, rule-based and machine learning approaches have been used to extract MTIs. Numerous public databases, such as miRCancer (43) and miRSel (46), have been constructed using rule-based approaches to extract miRNA associations from text. In contrast, TarBase uses AIIAGMT, a machine learning gene-mention tagger that is based on the conditional random field (CRF) approach (13). miRWalk includes an automated text-mining search pipeline for screening miRNA-target-related titles/abstracts in PubMed using a dictionary-based and regular expression string matching approach (17). The regular expression string matching approach is a rule-based approach that is widely used to perform NER. Rule-based approaches rely on explicit rules, which are not sufficiently flexible to cover all variations of patterns. Machine learning approaches learns patterns automatically, but the results are not comprehensible to humans.

Accordingly, this work developed an automatic text-mining module adopting a principle-based approach (PBA) (47) for the recognition of miRNA/target mentions and the extraction of miRNA and target gene interactions. PBA uses automatically extracted principles (so-called dominating patterns) and alignment matching to harness the advantages of both machine learning and rule-based approaches and to overcome their limitations. PBA is automatic and its results are comprehensible to human.

The proposed retrieval system has the following advantages over existing automatic methods for extracting miRNA targets; (i) based on an annotated training corpus (48), it constructs a principle-based model that rapidly screens abstracts across the entire PubMed database; (ii) it automatically downloads all open access papers in PubMed, as opposed to only those in the PubMed Center; (iii) it adopts a semantic model with PBA to improve performance and (iv) it supports miRNA and gene name recognition for a large set of species. Consequently, the developed retrieval system achieved an accuracy rate of 71.43%. All selected literatures in miRTarBase 6 are also validated through several curators. The Supplementary Methods include detailed results of evaluations of NER and relation extraction.

**Figure 2.**
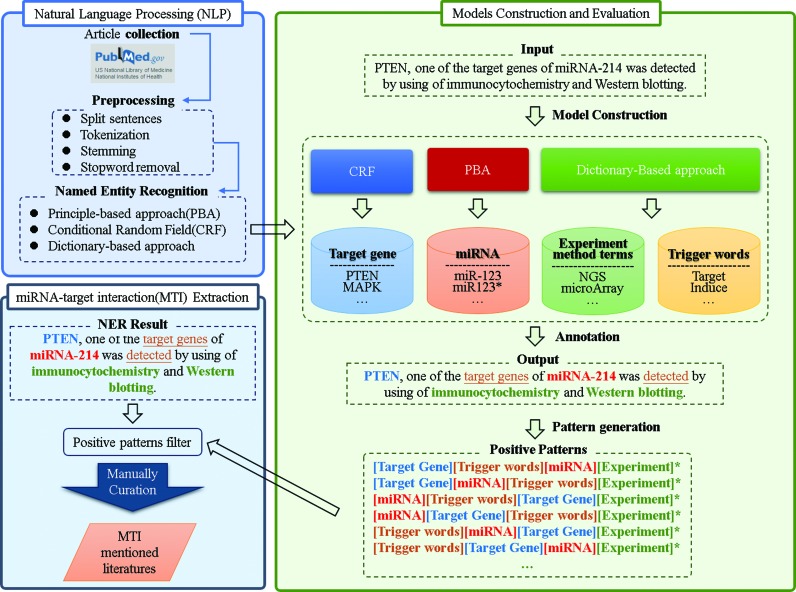
Natural language processing (NLP) techniques for finding MTI articles. (**i**) Articles collected from PubMed; (**ii**) preprocessing articles in the following steps; split sentences, tokenization, stemming and stopword removal; (**iii**) Named Entity Recognition (NER) based on principle-based approach (PBA), Conditional Random Field (CRF) and dictionary-based approach; (**iv**) following construction and evaluation of model, MTI extraction protocol was developed using PBA; (**v**) all curated articles were manually evaluated by biological domain experts.

Figure 2 displays workflow of the retrieval system. First, miRTarBase 6 extracts sentences from a previously mentioned annotated corpus (48) that contains miRNA and gene-related information. Then, a set of principle patterns is formed for each miRNA-gene pair in those sentences. Finally, these principle patterns are used to classify each miRNA–gene pair as either exhibiting miRNA-target interaction or not. This process is described in detail in the Supplementary Methods section.

### Enhanced web interface

Table 1 and Figure 1 show major improvements in web interface graphical visualization. TCGA miRNA-seq and RNA-seq expressions are integrated; the error report system is updated; and the search system improved. miRNA-disease information is displayed as word clouds, and the miRNA-target site viewer uses CLIP-seq data. The TCGA expression data display the correlation between the expression fold change of miRNA and mRNA to indicate miRNA direct targets. The miRNA-disease information word cloud provides more information about experimentally validated miRNA-associated diseases and the relationship between miRNA-target interactions and disease. Therefore, miRNAs could be used as biomarkers in disease diagnosis and provide clues concerning potential disease therapies.

#### Word cloud of miRNA-disease information

MicroRNAs play essential roles in different diseases as either tumor suppressors or oncogenes. Their expression pattern is tissue-specific and holds potential for therapeutic targets and novel biomarkers. The word cloud of miRNA-disease information is improved in the updated version of miRTarBase. A user who searches for a microRNA of interest in miRTarBase can now learn about its association with different diseases. The cloud of disease-related words provides at a glance useful information about the role of microRNA in diseases. For example, hsa-miR-122–5p is the microRNA that is most frequently found in liver and associated with liver biology and liver diseases such as hepatitis and hepatocellular carcinoma (Figure 1B).

#### miRNA-Target site viewer in CLIP-seq data

CLIP is a method that combines ultraviolet (UV) cross-linking with immunoprecipitation to identify specific protein–RNA interactions. Large-scale MTIs have been recently identified using the CLIP-seq approach including HITS-CLIP, PAR-CLIP and CLASH. The first step in CLIP-seq data processing is to map all of the reads to the genome and transcriptome; it is followed by cluster detection and, finally, binding site detection (49). miRTarBase 6 provides evidence of the interaction between has-miR-15a-5p and the VEGFA target gene by CLIP sequencing that was collected from 17 data sets and validated by studies that include luciferase assays (50–52). Executing this interaction of the microRNA-target gene in the CLIP-seq viewer interface has intensified the quality of the updated version of miRTarBase. Figure 1C presents the information in the data sets and the miRNA–mRNA target sites that are obtained by analysis of the CLIP-seq data.

#### Clinical microRNA and gene expression profiles from TCGA

TCGA collects large clinical data sets to develop microRNA biomarkers and to help biologists identify tumor-specific miRNA regulatory pathways. The correlation between the expression values of miRNA and mRNA provides an important indication of direct targets of miRNA. For instance, the change in miR-122–5p and ALDOA reveals the negative correlation significantly only in human hepatocellular carcinoma (*P*-value = 3.9e−7 and R = −0.639) (Figure 1D and Supplementary Figure S2). Experimentally validated data reveal that miR-122 suppresses tumors and targets ALDOA in hepatocellular carcinoma (45).

## RESOURCES THAT INCORPORATE MIRTARBASE AND ITS APPLICATIONS

Recently, many researchers have utilized miRTarBase. Four main applications of this database are summarized here. The first is in the elucidation of miRNA functions under different conditions and in different species. Notably, miRTarBase is being utilized to determine a molecular mechanism of non-coding RNAs in cancer progression (53); to find novel biomarkers for endometrial carcinoma (54) and to integrate microRNA regulatory networks in rat kidney and mouse liver (55,56). Many research studies have used miRTarBase to extend related findings. For instance, miRTarBase has been used for the *in silico* identification of confirmed targets of miRNA of gingival tissue in periodontitis in cases of obesity (57). Key microRNAs in coronary artery disease have been identified by using miRTarBase (58). A validated data set from miRTarBase has been used to identify genes that are targeted by miRNAs of interest during desmoid tumor progression (58). The second application of miRTarBase is to provide a training set for the development of microRNA target prediction algorithms that use machine learning. Conventional prediction tools have been developed based on many biological features of miRNA/target duplexes, including seed match type, the minimum free energy (MFE) of the MTI and structural characteristics. These tools provide improved predictive accuracy for one class of miRNA-target interactions but low accuracy for others. Recently, the predictive power of some miRNA prediction tools (59,60) has been improved by considering more features of experimentally verified MTIs in miRTarBase. In that case, information about additional biological features of miRNA/target duplex from carefully curated MTI data sets is used to develop new miRNA target predictors. The third application is to provide a benchmark data set that can be used to evaluate the performance of different miRNA target prediction tools. The identification of MTI candidates is critical to determining functional roles of the many miRNAs. Finally, miRTarBase is an experimentally validated collection of miRNA-target interactions and is the more updated collection of MTIs, as its content is continually compared with that of previously developed databases. Ensembl (61), Mouse Genome Database (MGD) (62) and GeneCards (63) are comprehensive databases of genomic, transcriptomic, proteomic and functional information about known or predicted genes; they incorporate MTIs from miRTarBase. miRTarBase has also been incorporated into an increasing number of miRNA resources such as miRBase (8), TarBase (13), miRCancer (43), miRWalk (17), miRGator (10), miRGate (19), starBase (44), HMDD (14) and others.

## CONCLUSIONS AND PERSPECTIVES

This work presents a more comprehensive collection of experimentally validated miRNA-target interactions. The current update has seven times as many MTIs than the miRTarBase 4.5 release, and adds a significant number of MTIs that were collected from high-throughput experimental methods, such as CLIP-seq and PAR-CLIP. This version integrated new features, such as NLP, to recognize miRNA-target interactions between miRNAs and their target genes. Additionally, it provides information on the up- or down-regulation of miRNA and its target gene using TCGA. Abstract annotation data are available for download.

The latest release of miRTarBase includes 366 181 curated miRNA-target interactions between 3786 miRNAs and 22 563 target genes, collected from over 4966 articles. The PBA was used to recognize miRNAs in the database, achieving an F-score of 98.7% on a manually curated test data set. The PBA outperformed the traditional rule-based method by 5.2%. The conditional random method was utilized for target gene recognition, achieving an F-score of 70%. For the extraction of MTIs, a set of patterns was automatically generated by the PBA. Each pattern comprises a miRNA, its target gene and other key concept terms, such as those pertaining to unique experimental methods and trigger words. Using a screening mechanism that is based on NLP, the system identified 854 papers that may provide a summary of MTIs. Manual proofreading confirmed that 610 papers mentioned MTIs, indicating that the NLP technique herein had an accuracy of 71%. An automatically updated pipeline was constructed to perform monthly updates that provide the latest MTI information. In summary, miRTarBase 6 serves as a repository of extensive experimental information of value for a wide range of miRNA-related research. miRTarBase will be continuously maintained and updated. The regular update involves manual curation of miRNA-target interactions, and integration of TCGA RNA-seq and miRNA-seq data, providing more comprehensive gene and miRNA expression data across various tumors and samples.

## AVAILABILITY

The database is now publically accessible through the URL http://miRTarBase.mbc.nctu.edu.tw/.
